# Design and Modeling of a Device Combining Single-Cell Exposure to a Uniform Electrical Field and Simultaneous Characterization via Bioimpedance Spectroscopy

**DOI:** 10.3390/s23073460

**Published:** 2023-03-25

**Authors:** Rémi Bettenfeld, Julien Claudel, Djilali Kourtiche, Mustapha Nadi, Cyril Schlauder

**Affiliations:** Institut Jean Lamour, CNRS, Université de Lorraine, F-54000 Nancy, France; remi.bettenfeld@univ-lorraine.fr (R.B.);

**Keywords:** biosensor, single cell, microelectrode, impedance spectroscopy, electric exposure

## Abstract

Previous studies have demonstrated the electropermeabilization of cell membranes exposed to an electric field with moderate intensity (<2 V/cm) and a frequency of <100 MHz. Bioimpedance spectroscopy (BIS) is an electrical characterization technique that can be useful in studying this phenomenon because it is already used for electroporation. In this paper, we report a device designed to perform BIS on single cells and expose them to an electric field simultaneously. It also allows cells to be monitored by visualization through a transparent exposure electrode. This device is based on a lab-on-a-chip (LOC) with a microfluidic cell-trapping system and microelectrodes for BIS characterization. We present numerical simulations that support the design of the LOC. We also describe the fabrication of the LOC and the first electrical characterization of its measurement bandwidth. This first test, performed on reference medium with a conductivity in the same order than human cells, confirms that the measurement capabilities of our device are suitable for electrical cells characterization.

## 1. Introduction

Clinical and in vitro studies have suggested that exposure of living cells to an electric field can produce nonthermal antiproliferative effects at frequencies ranging from a few kilohertz to hundreds of megahertz [1,2,3,4]. These effects may be the result of a modification of the ion flux through the cell membrane which increases its permeability to ions and molecules [5]. This electropermeabilization can occur at relatively low levels of electric field intensity. For example, Wust et al. exposed human cancer cell lines HT-29 and SW480 to an electric field of 2 V/cm at 13.56 MHz, and reported evidence of nonthermal effects on their proliferation [3]. Loghavi et al. described the electropermeabilization of bacterial membranes (*Lactobacillus acidophilus*) exposed to a moderate-intensity electric field (about 2 V/cm) at 45 and 60 Hz and reported that cell growth was impacted [6]. Therefore, this type of exposure should be further investigated over a larger range of frequencies and intensities. In this context, some related electroporation studies have already employed some intriguing tools. Electroporation causes the extreme electropermeabilization of cell membranes by exposing them to short-duration, high-intensity electric pulses. Biochemical models and molecular simulations suggest that this is due to aqueous pores forming in the membranes, as well as chemical modifications to their lipids and proteins [7]. Some researchers have used microelectrodes to obtain bioimpedance spectroscopy (BIS) measurements of cells after or during electroporation.

BIS is a technique for characterizing the electrical impedance of biological samples [8]. In particular, BIS of cells can provide a signal dependent on the properties of their constituent materials and dimensions, and it can achieve this in a high-throughput manner while preserving cell integrity [9]. Electropermeabilization of a cell membrane changes its electrical properties. Because ions can pass through the membrane more easily, its electrical resistance decreases, and its permeability to electric current increases. Therefore, BIS might be an excellent technique for monitoring electroporation and, potentially, electropermeabilization. García-Sánchez et al. exposed three cell lines to high-intensity (>600 V/cm) electric pulses applied into the biological medium via concentric electrodes [10]. The same electrodes were used to characterize cells using BIS during the intervals between pulses. Similarly, Shamaee et al. created a device that used interdigital electrodes placed in the biological medium to study the exposure of human cancer cell line MDA-MB-231 to a low-intensity electric field (about 1.7 V/cm) at 150 kHz [11]. They found that exposure to the field reduced cell growth and affected cell impedance. One problem with the devices used in these two studies arises from the nonuniformity of the cells’ exposure. The electric exposure field was nonuniform because it was applied to hundreds of cells simultaneously, raising the possibility that some cells might mask the field from others. Another limitation of these devices is that they could not simultaneously expose cells and characterize them by BIS. In this study, to overcome the limitations mentioned above, we propose an approach based on an original lab-on-a-chip (LOC) integrated in an exposure device. This LOC includes microfluidic trapping channels and microelectrodes. The latter are used to interrogate the impedance of single cells, isolated in the channels, by BIS. BIS using LOCs is a technique now employed in many research fields including cancer, neurodegenerative diseases and toxicology, as reported by Hassan et al. [12]. It has been used to monitor the viability of cells [13], to specify their differentiation [14], and to analyze the evolution of a cell culture [15]. One advantage of using an LOC is that it enables single-cell analysis by means of cell-trapping techniques. Of these, hydrodynamic trapping is the simplest method to fabricate and operate [16,17]. We chose this method for our device because of these advantages, and also to avoid the exposure of cells to another electric or magnetic field, as occurs in dielectrophoresis and magnetophoresis, respectively.

We placed our LOC between exposure electrodes that produced an electric field. This ensured that the electrodes for exposure and BIS were uncorrelated and that the device could expose cells to the electric field and characterize them simultaneously. Our LOC had ten pairs of microelectrodes with a trapping area for each one so that ten cells can be characterized in each experiment. The microfluidic channels and the microelectrodes were designed for cells which are approximately 20 µm in diameter. We plan to use our device to characterize human cancerous monocytes THP-1 in our future studies. These somatic cells were used for cytotoxicity assays in our laboratory [18], and other studies have already performed BIS on these cells with success [19,20]. In this paper, we first present the device’s principal and characteristic dimensions. Next, we use numerical simulations to validate its design, simulating the exposure field, BIS measurement, and stream in a microfluidic trap. We then describe the fabrication and electrical characterization of the LOC component. Finally, we conclude by describing the utilization of the device in future studies.

## 2. Materials and Methods

### 2.1. Device Design

#### 2.1.1. Global Structure

**Figure 1:**
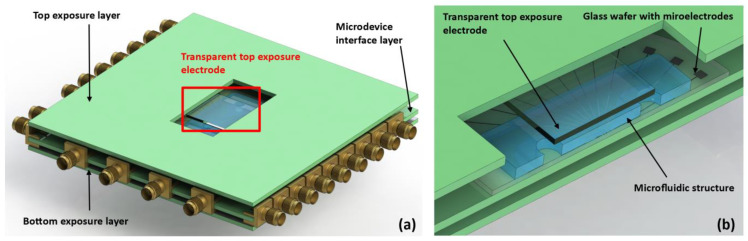
(**a**) A scaled 3D model of the device. (**b**) A cutaway view of the 3D model’s center showing the microelectrodes for BIS interrogation and the microfluidic channels.

Our device was divided into three layers superposed in parallel (Figure 1a,b). The top and bottom layers constituted the exposure electrodes, and the middle layer constituted the LOC, with microfluidic channels and microelectrodes for BIS measurement. In this context, the electric field in the middle layer of the device was quasi-uniform with a strength of E=V/d, where V is the applied voltage between the exposure electrodes and d is the distance between them. The middle layer was 1 cm thick, so that a field of ≤50 V/cm could be achieved without high voltages. The top exposure electrode was transparent, so that we could visualize cells and monitor microfluidic operations with an optical microscope.

The middle layer comprised a circuit board supporting a 2″ glass wafer on which the LOC was maintained. This circuit board had tracks printed on it, providing the connection between the microelectrodes and SubMiniature version A (SMA) connectors to link with the impedance analyzer. Spring contacts ensured electrical contact between the microelectrodes and the printed tracks without the need for soldering. The glass wafer was secured to the board with a 3D-printed component.

#### 2.1.2. Microelectrodes

**Figure 2:**
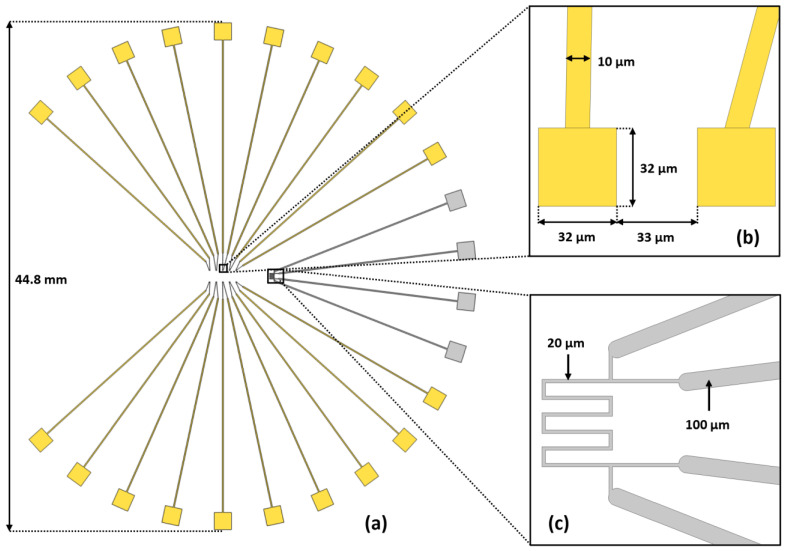
Schematic of the microelectrodes for BIS. (**a**) A global schematic: microelectrodes and their tracks are in yellow; the thermal sensor and its tracks are in gray. (**b**) A sensitive extremity of a microelectrode pair. The square extremity is 32 µm per side, and the SiO_2_ isolation layer covers a 1 µm perimeter of the Pt, making the final contact area with the biological medium 30 µm per side. (**c**) The temperature sensor makes in a Pt track.

Ten pairs of microelectrodes (Figure 2a) and their microfluidic traps were deposited on the glass wafer. Therefore, ten cells could be simultaneously characterized during one exposure, and a device with a deficient trap could still be used. The microelectrode design was based on the human macrophage characterization of Alves de Araujo et al. [21]. Because these cells have a medium diameter of about 20 µm, the sensitive part of the microelectrodes was of similar dimensions (30 µm square). This slightly larger size relative to the cells was needed to allow for uncertainty in the trapped cell’s position. They were made from platinum (Pt) due to its good electrical conductivity and biocompatibility. The tracks on the glass wafer were covered with a layer of silicon dioxide (SiO_2_) to avoid their contact with the biological medium and parasitic currents, which could have limited the measurement range. This layer formed a 1 µm perimeter around the sensitive part of the microelectrodes (Figure 2b). It was important to ensure that the sensitive area was precisely the same on all microelectrodes, even with the uncertain alignment between the Pt and SiO_2_ patterns. A temperature sensor was placed in the microfluidic channels’ output, comprising a track of Pt with a resistance of about 100 Ω (Figure 2c). The sensor was entirely covered by a SiO_2_ layer.

#### 2.1.3. Microfluidic Channels

**Figure 3:**
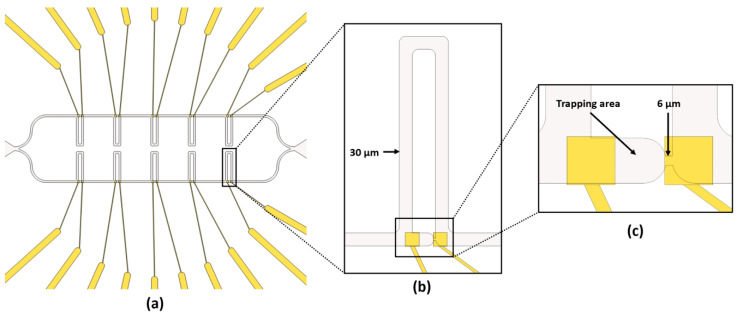
(**a**) Schematic view of the microfluidics channels (in gray) and microelectrodes for BIS interrogation (in yellow). (**b**) Two channels of a cell trap on a microelectrode pair. (**c**) The shorter channel of the microfluidic trap on a microelectrode pair when the cell becomes trapped.

The microfluidic component of the LOC comprised two parallel channels with five traps on each (Figure 3a). The channels had a square section with sides of 30 µm. The trap design was based on a model developed by Tan and Takeuchi [22]. Two parallel channels were used (Figure 3b). The shorter channel had a lower hydraulic resistance than the longer channel and narrowed to 6 µm at its end. Cells were therefore guided by the biological medium stream into the short channel, shown in Figure 3c, but were then blocked by its narrowing width. When a cell was blocked, it was then maintained in place by the difference in pressure between the two ends of the shorter channel. Hydraulic resistance was greater in the shorter than in the longer channel. Therefore, other cells continued their path in the longer channel.

### 2.2. Numerical Simulations

All simulations were performed using the finite element method (FEM) in COMSOL Multiphysics. The electrical properties of the materials used are summarized in Table 1. Cell’s electrical properties where chosen using classical values find in literature, as discussed in Section 2.2.2. “Microelectrodes and cell modeling”. Except for the extracellular medium, these values are average values and not a characterization of materials used for the fabrication of the device.

#### 2.2.1. Exposure Field

**Figure 4:**
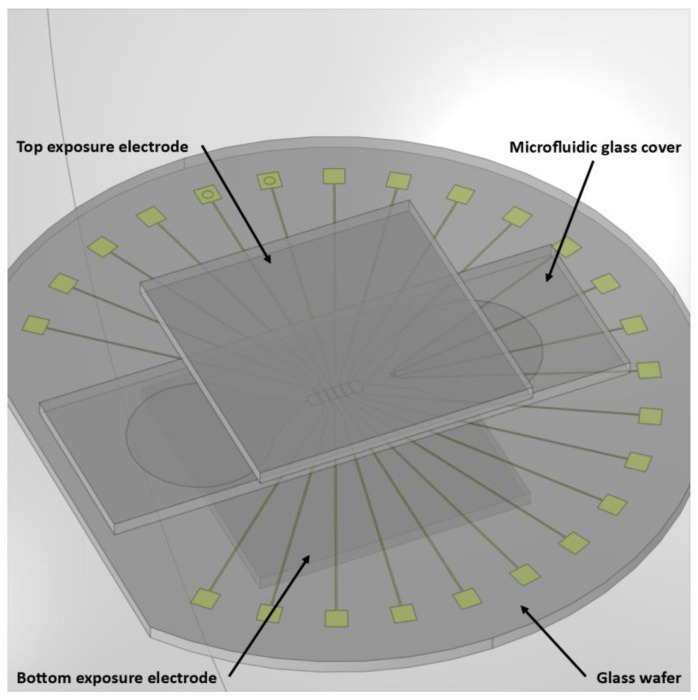
Device model in the FEM simulation.

The first simulation aimed to check the uniformity of the exposure field and used the model shown in Figure 4, comprising the microelectrodes, microfluidic channels, their glass wafer support, and the two exposure electrodes. We did not simulate the SiO_2_ isolating layer, circuit boards, or the 3D-printed component that secured the glass wafer. An air sphere constituted the volume of the simulation. The simulated dimensions of all elements were the same as their real dimensions presented in Section 2. All glass components (wafer, microfluidic cover, and glass support for the exposure electrodes) were 1 mm thick. The microelectrodes were fabricated in a Pt layer 120 nm thick (See Section 3). However, for simplicity, the two cases were tested for a voltage amplitude of 1 V between the exposure electrodes at frequencies of 1 kHz, 10 kHz, 100 kHz, and 100 MHz.

**Figure 5:**
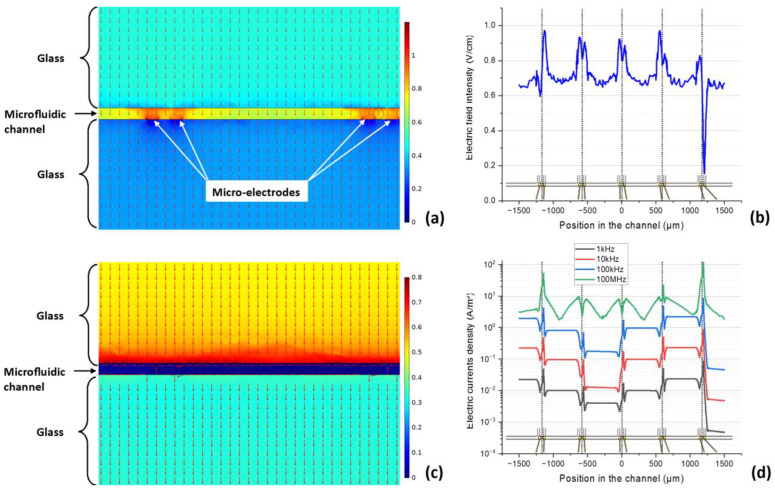
(**a**) Vertical cutaway of the simulated device in the middle of a microfluidic channel exposed to an electric field of 1 MHz and 1 V between the exposure electrodes. The colors correspond to the electric field intensity (V/cm) and the arrows to its direction. (**b**) Electric field intensity along the middle of the microfluidic channel. Vertical dotted lines correspond to the cell-trapping positions. The intensity peaks are caused by the five microelectrode pairs disturbing the field. (**c**) The same cutaway view as in (**a**), with exposure to an electric field of 1 MHz and 1 V between the exposure electrodes, but when the channel is full of an extracellular medium. (**d**) Electrical current density along the middle of the microfluidic channel at different frequencies. Vertical dotted lines correspond to the cell-trapping positions. Higher frequencies are more absorbed by the biological medium.

**Figure 6:**
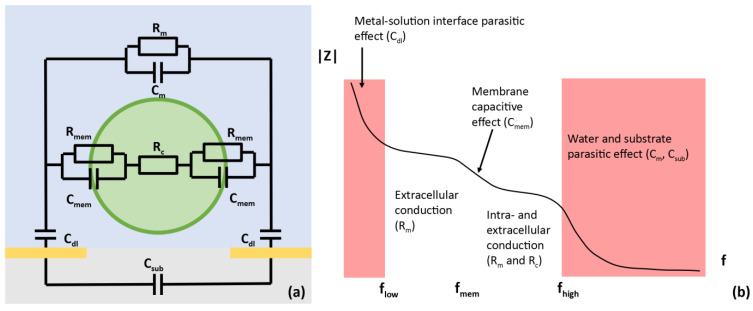
(**a**) The electrical model of a cell (green) inside an extracellular medium (blue) between two electrodes (yellow) on their substrate (gray). (**b**) The typical impedance spectrum of a cell is limited by the parasitic effects of the electrodes and the environment.

In the first case, the microfluidic channels were full of air, and the obtained field (Figure 5a,b) was applied to the biological sample (cell and extracellular medium). The cells were exposed to an electrical field of between 0.5 and 0.95 V/cm, even if the microelectrodes disturbed it. In the second case, shown in Figure 6, the microfluidic channels were full of material with electrical properties typical of an extracellular medium (Table 1). Therefore, this simulation represented the distribution of currents inside a device without cells. As could be anticipated, given the electrical properties of water, fields with higher frequencies were better absorbed and generated higher currents.

#### 2.2.2. Microelectrodes and Cell Modeling

The cell membrane’s electrical impedance was modeled using a standard Fricke’s model (Figure 6a). Cell membranes have a characteristic impedance signature that can be masked by the parasitic effects of the microelectrodes performing the measurement and their environment. Figure 6b shows a typical impedance spectrum of a cell between two electrodes. The lower frequency limit of the measurement is due to the interaction between the medium and the microelectrodes’ surface that doubles the layer capacity. At high frequencies, the capacitive interaction between the two electrodes (mainly between their tracks) causes another capacitive effect. Between these limits, it is possible to observe the capacitive effect of the cell membrane itself, and also the conductive effects of intra and extracellular media (two plateaus). The passage from first to second plateau is called Beta dispersion and correspond to electrical membrane effect. To correctly characterize biological tissues or single cells it is necessary to have a sufficient measurement bandwidth centered to the frequency of Beta dispersion. This bandwidth depends on the biological medium conductivity, the electrodes’ geometry, the environmental material, and the cell’s properties. Frequency of Beta dispersion only depends on biological medium properties (intra-extracellular medium, membrane capacitance and cell size) as described in models such as Maxwell mixture theory or Cole-Cole and already discussed in a previous work [21]. Characteristic conductivities and frequency of Beta dispersion for different human tissues can be find in the reference database based on Gabriel’s work [22]. For example, typical conductivity of blood cells sample is varying around 1 S/m with a Beta dispersion frequency about 1 MHz, as chosen for our simulations. Microelectrodes induce high levels of measured impedance (typically hundreds of ohms) which make them very sensitive to the capacitive effects of tracks. The numerical simulations which we now describe were needed to ensure that our device had sufficient bandwidth for the type of cells used in the study. This is one of the most important aspects of the design of sensors based on electrical impedance measurements.

**Figure 7:**
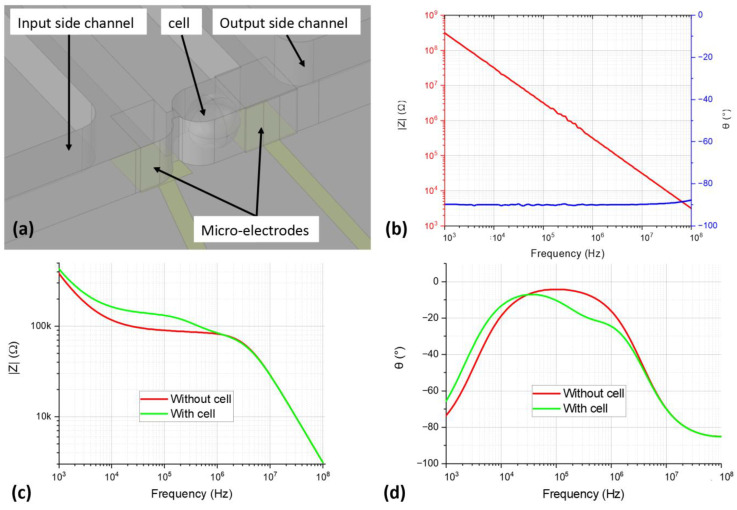
(**a**) A zoomed view of a 3D model of the simulated device’s trapping area with a cell model in the channel. (**b**) The impedance of a microelectrode pair in the SIM_AIR simulation. (**c**) A comparison of the impedance modulus of a microelectrode pair in the SIM_SOL and SIM_CELL simulations. (**d**) A comparison of the impedance phases of a microelectrode pair in the SIM_SOL and SIM_CELL simulations.

Simulations of three cases of impedance measurements were performed to characterize the device and to ensure that it was suitable for BIS cell measurements. The device’s model was the same as that presented above. In the first simulation (SIM_AIR), the microfluidic channel was full of air, so that an impedance measurement for an open device could be obtained. In the second simulation (SIM_SOL), the channel was full of an extracellular medium. In the third simulation (SIM_CELL), a cell model was placed between a pair of microelectrodes (Figure 7a). In all three cases, an interrogation signal was applied to the extremity opposite the cell of one of the two electrodes in a frequency range from 1 kHz to 10 MHz. The cell model consisted of a sphere full of the same material as the extracellular medium. The membrane was a surface condition with electrical properties calculated from the material properties (Table 1) and a thickness of 10 nm.

The SIM_AIR simulation results (Figure 7b) show the expected capacitive effect between the electrodes. The impedances obtained in the SIM_SOL and SIM_CELL simulations are compared in Figure 7b,c. The parasitic effects of the double-layer capacitance and the extracellular medium’s permittivity are visible, as is the membrane impedance signature centered at 300 KHz in the bandwidth from 20 kHz to 2 MHz. These results indicate that the microelectrode design was suitable for performing BIS on a cell of about 20 µm diameter with specific conductivity around 1 S/m as human monocytes. A similar impedance was measured on THP-1 cells in the work of Turcan et al. and Messer et al. [19,20].

#### 2.2.3. Microfluidic Streams

**Figure 8:**
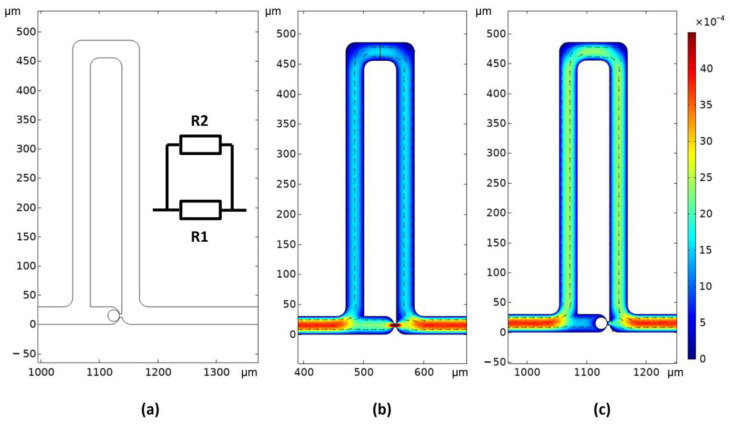
(**a**) Schematic of the microfluidic channel model and an associated diagram showing hydrodynamic resistances R1 (narrow channel and trapping area) and R2 (bypass channel). (**b**,**c**) Cutaway views of fluid velocity (in m/s) in a channel (**b**) before and (**c**) after cell trapping. Note that R1 > R2 in (**b**) and R1 < R2 in (**c**).

The authors of the cell-trap model reported that the hydraulic resistance in the trapping channel must be lower than the resistance in the bypass channel (Figure 8a). In this study, therefore, the fluid stream in the channels was simulated with and without a cell in the trap. The simulation followed the Hagen–Poiseuille law, and the cell model was a 20 µm rigid sphere. An input flow rate of 0.1 µL/min was imposed. Figure 8b shows a cutaway view through the middle of the channel with the resulting fluid velocity. Because the cross section was the same for all channels except the narrow channel beyond the trapping area, the fluid velocity was proportional to the flow rate. Therefore, the flow rate was higher in the trapping area before a cell was trapped and lower after a cell was trapped, as illustrated in Figure 8b,c. Consequently, we sought to guide cells into the trapping area where they will be immobilized.

## 3. Results

### 3.1. Transparent Top Exposure Electrode

**Figure 9:**
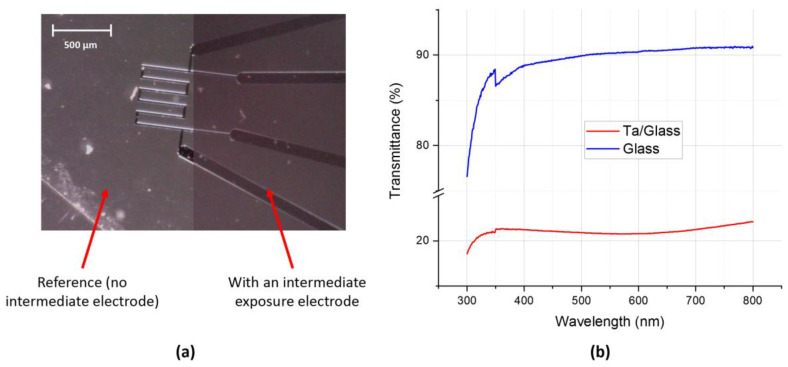
(**a**) Microscope observations of the thermal sensor without (left) and with (right) the transparent exposure electrode above them. (**b**) The optical transmittance spectra of the transparent exposure electrode (Ta/glass) and the glass substrate (glass).

The top exposure electrode was made of a thin tantalum (Ta) layer (17 ± 2 nm), which was deposited via a phase vapor deposition process (PVD) onto a glass surface 1 mm thick. This was sufficiently transparent to allow for the visualization and the monitoring of cells through the electrode using an optical microscope. Figure 9a shows the thermal sensor viewed under the top exposure electrode with a microscope for the demonstration. The transmittances of the glass substrate alone and with the Ta layer were evaluated using an Agilent Cary 7000 UMS spectrophotometer (Figure 9b).

### 3.2. Microelectrodes

**Figure 10:**
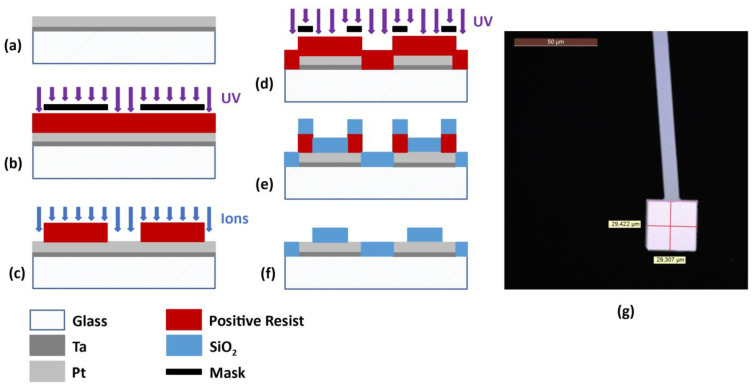
The main microelectrode fabrication steps. (**a**) Deposition of Ta (10 nm) and Pt (110 nm) layers on a glass substrate using a PVD process. (**b**) Positive resist deposition and insolation through a mask. (**c**) Resist development and IBE of Pt and Ta. (**d**) Deposition and insolation of a positive resist for lift-off. (**e**) Resist development and deposition of SiO_2_ layer (120 nm) using a PVD process. (**f**) Resist lift-off. (**g**) Image of a microelectrode. The lighter square area corresponds to the sensitive surface not covered by SiO_2_.

Figure 10 illustrates the main steps in fabricating the microelectrodes for BIS interrogation. They were created in a 110 nm thick Pt layer deposited on a 2″ borosilicate glass wafer using a PVD process. A thin (10 nm) Ta layer was required between the Pt and the glass because of their poor adhesion. The pattern was structured by photolithography and ion beam etching (IBE). A 120 nm thick SiO_2_ layer was deposited by PVD and patterned using a lift-off process.

### 3.3. Microfluidic Channels

**Figure 11:**
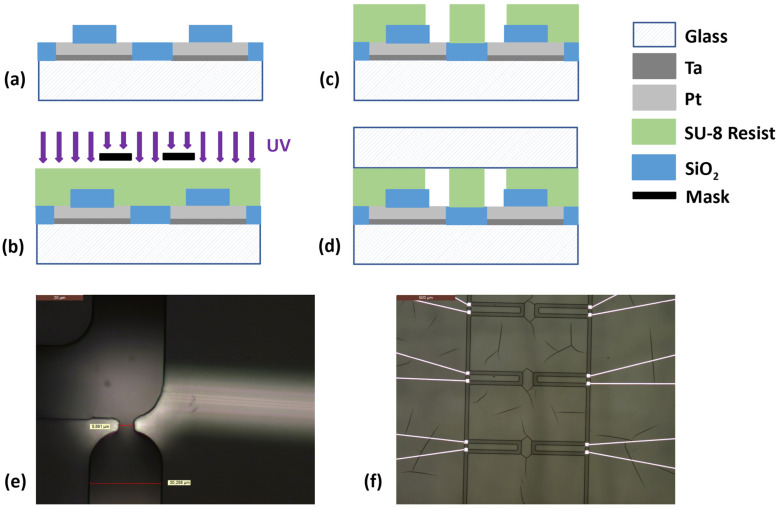
The main microfluidic structure fabrication steps. (**a**) The previously fabricated microelectrodes. (**b**) SU-8 deposition and insolation. (**c**) SU-8 development. (**d**) The bonding of a piece of glass to the SU-8 to close the channels. (**e**) An image of the narrow channel of a microfluidic trap. (**f**) A view of the microfluidic channel over the microelectrodes.

Figure 11 illustrates the main steps in fabricating the microfluidic structure on the microelectrodes. The walls of this structure were made of SU-8 2015 photonegative resist. This epoxy-based resist is suitable for making functional structures and can be patterned by photolithography. The SU-8 layer was 30 µm thick, in order to obtain a square channel cross section. The channels were closed with a piece of glass of 1 mm thickness which was bonded to the SU-8 during its hard bake.

### 3.4. Impedance Characterization

The fabricated microelectrodes were compared with their numerical models by means of an impedance characterization; this was required to confirm that the device had sufficient bandwidth for the analysis of cell with a specific conductivity about 1 S/m. The simplification in the numerical models meant that we could not rely only on these entirely. We performed two measurements using a Keysight Technologies E4990A impedance analyzer (Santa Rosa, CA, USA): one with an empty channel (MEAS_AIR), and another with a conductivity reference solution of 1.167 S/m (MEAS_SOL). To compensate for the impedance of the cables and the circuit board used to interface the LOC with the impedance analyzer, which were not simulated, an open measurement with the interface only was taken. The compensated impedance of a pair of microelectrodes $Z_{elec}$ was calculated in terms of the open impedance $Z_{open}$ and the impedance with the LOC $Z_{m}$ by the following equation:

(1) $$Z_{elec}=\frac{Z_{m}\times Z_{open}}{Z_{m}+Z_{open}}.$$

**Figure 12:**
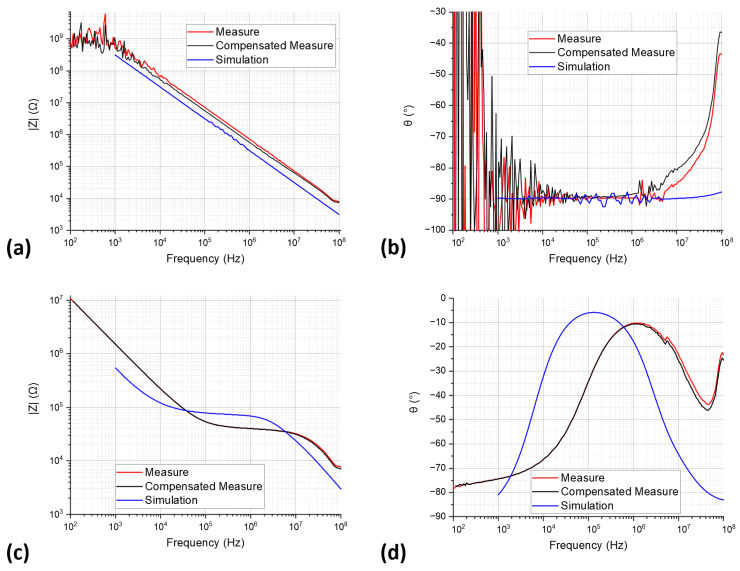
Comparison of the impedance modulus (**a**) and phase (**b**) of a microelectrode pair with a channel full or air measured (MEAS_AIR with and without compensation obtained with the Equation (1)) and simulated (SIM_AIR). (**c**,**d**) Comparison of the impedance modulus (**c**) and phase (**d**) of a microelectrode pair with a channel full of an ionic solution measured (MEAS_SOL with and without compensation obtained with the Equation (1)) and simulated (SIM_SOL).

Figure 12 presents a comparison of these measurements with the simulation results. Several significant differences can be observed, especially underestimation of the capacitive effects in the simulation for both cases, and inductive effects in the measurements at 1 MHz for MEAS_AIR, and 30 MHz for MEAS_SOL. The obtained bandwidth, from 100 kHz to 10 MHz, well covers the Beta frequency found in both the simulation and the literature.

## 4. Discussion

The simulations showed that the parallel electrodes exposing cells to a uniform electric field in our device caused some performance limitations. The microelectrodes required for the BIS measurement disturbed the field. However, this field was more uniform and unidirectional than fields obtained with coplanar electrodes, as in the study of Shamaee et al. [11], for example. The exposure field between two points of the cell trapping area did not vary by more than a factor of two.

The device simulations also showed that our design was generally well adapted to trap cells and perform BIS on them. Nevertheless, there were some differences between the fabricated and modelled LOCs, due to the properties of the materials involved, the simplifications in the model, and the tolerances of the photolithography method. This was especially the case in the smaller dimensions of the narrow channel. These findings will be taken into account when determining calibration data for future measurements. Consequently, it will also be necessary to verify the bandwidth of the real microelectrodes when future measurements are carried out. This was conducted by measuring the impedance values both when a channel was full of air and when it was full of a solution with a conductivity close to that of the cells’ medium following the method outlined by Claudel et al. [24]. The results of our measurements showed a higher impedance, by about a factor of two, in the case of MEAS_AIR, compared with SIM_AIR, a difference beneficial to the measurement of a cell impedance. In a similar way, the MEAS_SOL showed a bandwidth shifted towards higher frequencies so that cell impedance was more easily observed than in the simulation. Overall, the simulation can be seen as a good design tool for presenting the worst-case scenario.

## 5. Conclusions

In this study, we set out the principles of a device capable of performing BIS on ten single cells during their exposure to an electric field. The device allowed the monitoring of cells during experimentation through a partial transmittance in the visible spectrum of the top exposure electrode. The device’s capabilities in exposing cells to a uniform electric field and performing BIS on them were confirmed by numerical simulations. The LOC part of the device, with microelectrodes and microfluidic channels, was fabricated and its impedance was characterized by calibrated solution of conductivity in the same order as the biological samples. Though some differences were found between the simulated and fabricated microelectrodes, we obtained a suitable useful bandwidth. The first electrical tests with the fabricated LOC confirmed that the measurement bandwidth was sufficient for targeted living cell characterization. The dimensions of the microfluidic traps were also established with the help of a numerical simulation. Their performance will be further evaluated with synthetic microspheres and somatic cells in future studies.

The design of this device and its electrical validation represent a first main step in studying the effects of electric field exposure on the cell membrane by means of BIS of single cells. We now plan to set up a complete experimentation bench based on our device to characterize exposed THP-1 monocytes. We aim to study the effects of electric field exposure on the cell membrane at frequencies from 1 kHz to 100 MHz. However, the device can also be used with a greater range of frequencies. It is possible to perform BIS characterization on cells during electric field exposure when connected to a fast impedance analyzer, enabling the study of membrane electropermeabilization kinetics with a fine temporal resolution.

## Figures and Tables

**Table 1:** Electrical properties used in the simulations.

Material	Conductivity (S/m)	Permittivity (F)	Reference
Air	0	1	COMSOL
Glass	10^−14^	3.75	COMSOL
Extracellular medium	1.167	78	Reference solution (see Section 3.4)
Cell cytoplasm	1	78	[23]
Cell membrane	10^−13^	11.3	[23]
Platinum	8.9 × 10^6^	1	COMSOL

## Data Availability

Not applicable.

## References

[1] Jang Y., Lee W.S., Sai S., Kim J.Y., Kim J.-K., Kim E.H. (2022). Tumor-treating Fields in Combination with Sorafenib Restrain the Proliferation of Liver Cancer in Vitro. Oncol. Lett..

[2] Wu H., Yang L., Liu H., Zhou D., Chen D., Zheng X., Yang H., Li C., Chang J., Wu A. (2021). Exploring the Efficacy of Tumor Electric Field Therapy against Glioblastoma: An in Vivo and in Vitro Study. CNS Neurosci. Ther..

[3] Wust P., Kortüm B., Strauss U., Nadobny J., Zschaeck S., Beck M., Stein U., Ghadjar P. (2020). Non-Thermal Effects of Radiofrequency Electromagnetic Fields. Sci. Rep..

[4] Jimenez H., Wang M., Zimmerman J.W., Pennison M.J., Sharma S., Surratt T., Xu Z.-X., Brezovich I., Absher D., Myers R.M. (2019). Tumour-Specific Amplitude-Modulated Radiofrequency Electromagnetic Fields Induce Differentiation of Hepatocellular Carcinoma via Targeting Cav3.2 T-Type Voltage-Gated Calcium Channels and Ca2+ Influx. eBioMedicine.

[5] Wust P., Stein U., Ghadjar P. (2021). Non-Thermal Membrane Effects of Electromagnetic Fields and Therapeutic Applications in Oncology. Int. J. Hyperthermia.

[6] Loghavi L., Sastry S.K., Yousef A.E. (2009). Effect of Moderate Electric Field Frequency and Growth Stage on the Cell Membrane Permeability of *Lactobacillus Acidophilus*. Biotechnol. Prog..

[7] Kotnik T., Rems L., Tarek M., Miklavčič D. (2019). Membrane Electroporation and Electropermeabilization: Mechanisms and Models. Annu. Rev. Biophys..

[8] Stupin D.D., Kuzina E.A., Abelit A.A., Emelyanov A.K., Nikolaev D.M., Ryazantsev M.N., Koniakhin S.V., Dubina M.V. (2021). Bioimpedance Spectroscopy: Basics and Applications. ACS Biomater. Sci. Eng..

[9] Xu Y., Xie X., Duan Y., Wang L., Cheng Z., Cheng J. (2016). A Review of Impedance Measurements of Whole Cells. Biosens. Bioelectron..

[10] García-Sánchez T., Bragós R., Mir L.M. (2018). In Vitro Analysis of Various Cell Lines Responses to Electroporative Electric Pulses by Means of Electrical Impedance Spectroscopy. Biosens. Bioelectron..

[11] Shamaee A.-M., Saviz M., Solouk A., Abdolahad M. (2020). An In Vitro Electric Field Exposure Device with Real-Time Cell Impedance Sensing. Iran. J. Sci. Technol. Trans. Sci..

[12] Hassan Q., Ahmadi S., Kerman K. (2020). Recent Advances in Monitoring Cell Behavior Using Cell-Based Impedance Spectroscopy. Micromachines.

[13] Dervisevic E., Tuck K.L., Voelcker N.H., Cadarso V.J. (2019). Recent Progress in Lab-On-a-Chip Systems for the Monitoring of Metabolites for Mammalian and Microbial Cell Research. Sensors.

[14] Zhou Y., Basu S., Laue E., Seshia A.A. (2016). Single Cell Studies of Mouse Embryonic Stem Cell (MESC) Differentiation by Electrical Impedance Measurements in a Microfluidic Device. Biosens. Bioelectron..

[15] Pan Y., Jiang D., Gu C., Qiu Y., Wan H., Wang P. (2020). 3D Microgroove Electrical Impedance Sensing to Examine 3D Cell Cultures for Antineoplastic Drug Assessment. Microsyst. Nanoeng..

[16] Deng Y., Guo Y., Xu B. (2020). Recent Development of Microfluidic Technology for Cell Trapping in Single Cell Analysis: A Review. Processes.

[17] Nilsson J., Evander M., Hammarström B., Laurell T. (2009). Review of Cell and Particle Trapping in Microfluidic Systems. Anal. Chim. Acta.

[18] Safar R., Doumandji Z., Saidou T., Ferrari L., Nahle S., Rihn B.H., Joubert O. (2019). Cytotoxicity and Global Transcriptional Responses Induced by Zinc Oxide Nanoparticles NM 110 in PMA-Differentiated THP-1 Cells. Toxicol. Lett..

[19] Turcan I., Caras I., Schreiner T.G., Tucureanu C., Salageanu A., Vasile V., Avram M., Tincu B., Olariu M.A. (2021). Dielectrophoretic and Electrical Impedance Differentiation of Cancerous Cells Based on Biophysical Phenotype. Biosensors.

[20] Messer R.L.W., Tackas G., Mickalonis J., Brown Y., Lewis J.B., Wataha J.C. (2009). Corrosion of Machined Titanium Dental Implants under Inflammatory Conditions. J. Biomed. Mater. Res. B Appl. Biomater..

[21] Alves de Araujo A.L., Claudel J., Kourtiche D., Nadi M. (2019). Use of an Insulation Layer on the Connection Tracks of a Biosensor with Coplanar Electrodes to Increase the Normalized Impedance Variation. Biosensors.

[22] Tan W.-H., Takeuchi S. (2007). A Trap-and-Release Integrated Microfluidic System for Dynamic Microarray Applications. Proc. Natl. Acad. Sci. USA.

[23] Alves de Araujo A.L., Claudel J., Kourtiche D., Nadi M. (2019). Influence of Electrode Connection Tracks on Biological Cell Measurements by Impedance Spectroscopy. Sensors.

[24] Claudel J., Ngo T.-T., Kourtiche D., Nadi M. (2020). Interdigitated Sensor Optimization for Blood Sample Analysis. Biosensors.

